# Supplementary material for: Specialized astrocytes mediate glutamatergic gliotransmission in the CNS
Source: Nature. 2023 Sep 6;622(7981):120–9. doi: 10.1038/s41586-023-06502-w (PMC10550825; doi:10.1038/s41586-023-06502-w)

nature portfolio

https://doi.org/10.1038/s41586-023-06502-w

Supplementary information

Specialized astrocytes mediate glutamatergic gliotransmission in the CNS

In the format provided by the authors and unedited

Supplementary Information

Gel full scans for **figure 3b** and **extended data figures 4c; 10b**.

Figure3

b

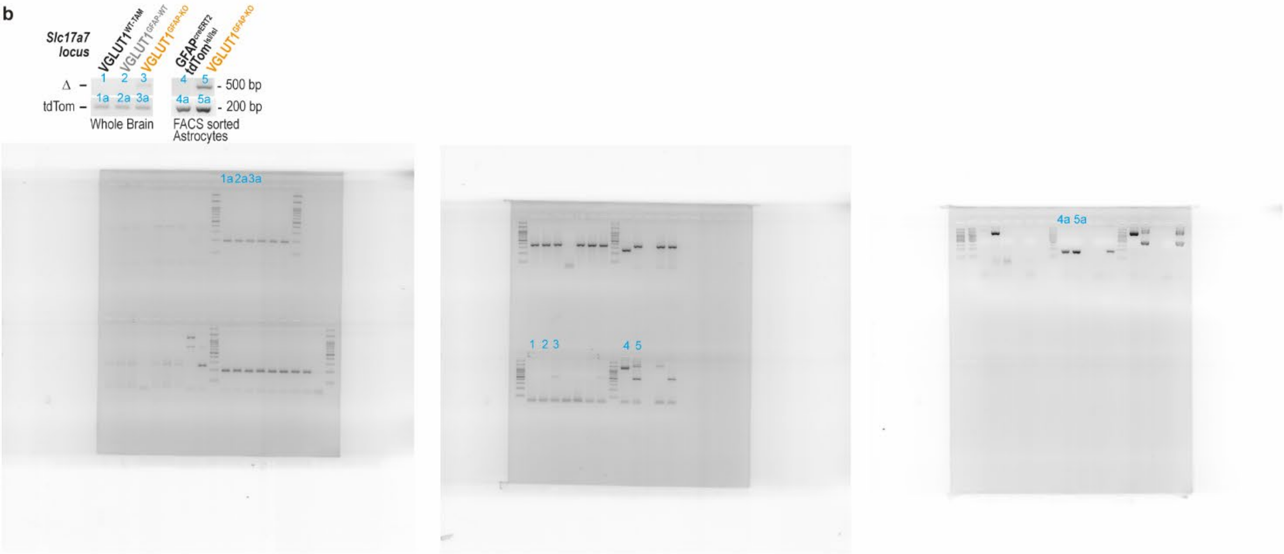

extended data 4

c

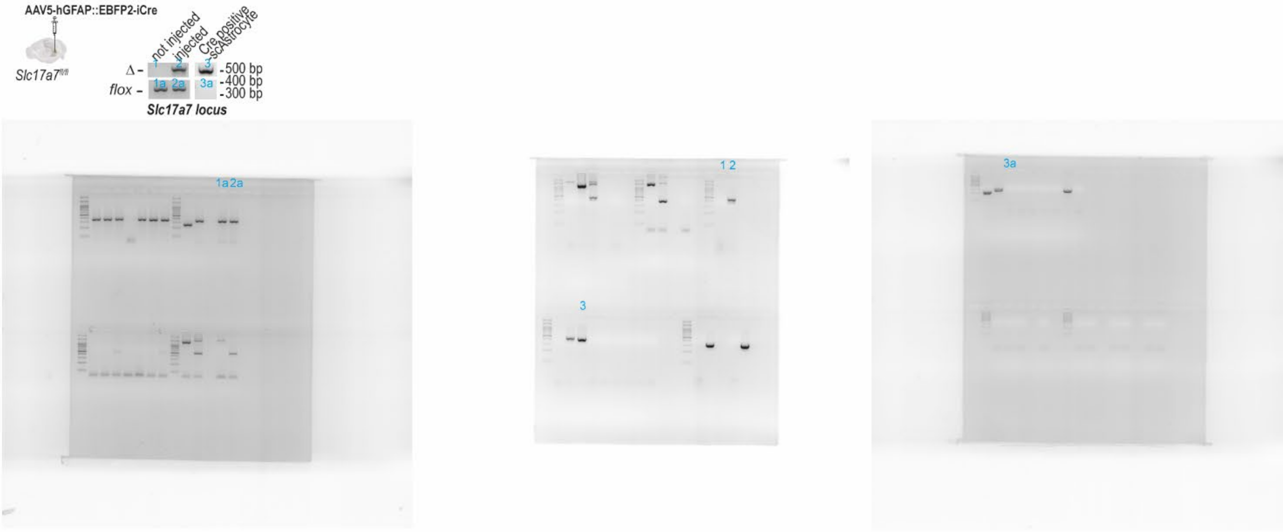

extended data 10

b

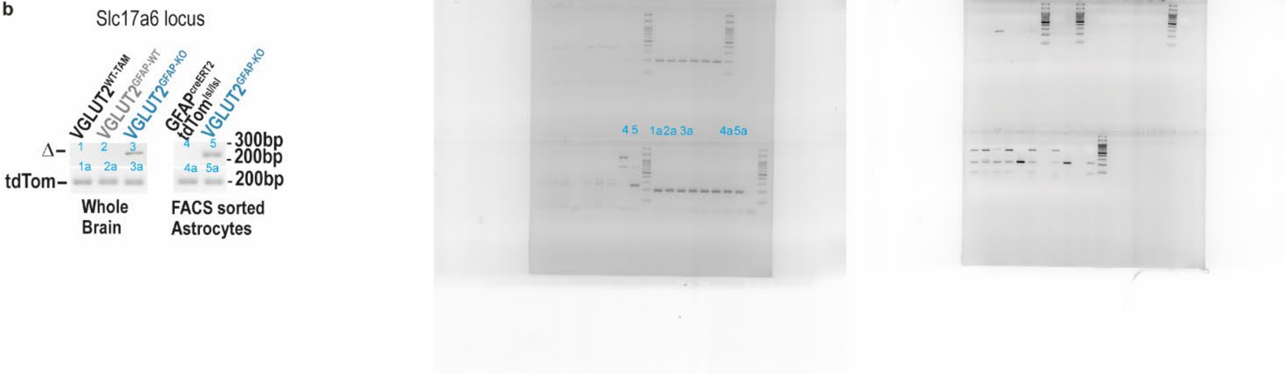

Supplement: Supplementary file 1 — Gel full scans for Fig. 3b and Extended Data Figs. 4c and 10b. [file 41586_2023_6502_MOESM1_ESM.pdf]
