# Supplementary material for: Specialized astrocytes mediate glutamatergic gliotransmission in the CNS
Source: Nature. 2023 Sep 6;622(7981):120–9. doi: 10.1038/s41586-023-06502-w (PMC10550825; doi:10.1038/s41586-023-06502-w)
Supplement: Supplementary file 2 — Reporting Summary [file 41586_2023_6502_MOESM2_ESM.pdf]

Corresponding author(s): Andrea Volterra and Ludovic Telley
Last updated by author(s): Jun 26, 2023

Reporting Summary

Nature Portfolio wishes to improve the reproducibility of the work that we publish. This form provides structure for consistency and transparency in reporting. For further information on Nature Portfolio policies, see our Editorial Policies and the Editorial Policy Checklist.

Statistics

For all statistical analyses, confirm that the following items are present in the figure legend, table legend, main text, or Methods section.

n/a	Confirmed	
☐	☒	The exact sample size (*n*) for each experimental group/condition, given as a discrete number and unit of measurement
☐	☒	A statement on whether measurements were taken from distinct samples or whether the same sample was measured repeatedly
☐	☒	The statistical test(s) used AND whether they are one- or two-sided<br>*Only common tests should be described solely by name; describe more complex techniques in the Methods section.*
☒	☐	A description of all covariates tested
☐	☒	A description of any assumptions or corrections, such as tests of normality and adjustment for multiple comparisons
☐	☒	A full description of the statistical parameters including central tendency (e.g. means) or other basic estimates (e.g. regression coefficient) AND variation (e.g. standard deviation) or associated estimates of uncertainty (e.g. confidence intervals)
☐	☒	For null hypothesis testing, the test statistic (e.g. *F*, *t*, *r*) with confidence intervals, effect sizes, degrees of freedom and *P* value noted<br>*Give P values as exact values whenever suitable.*
☒	☐	For Bayesian analysis, information on the choice of priors and Markov chain Monte Carlo settings
☒	☐	For hierarchical and complex designs, identification of the appropriate level for tests and full reporting of outcomes
☒	☐	Estimates of effect sizes (e.g. Cohen's *d*, Pearson's *r*), indicating how they were calculated

Our web collection on statistics for biologists contains articles on many of the points above.

Software and code

Policy information about availability of computer code

Data collection

Data collection, such as image acquisitions, electrophysiological recording etc. were performed with the specific instrument softwares installed on the instruments, as detailed in the methods.

Data analysis

Data analysis was performed with the following softwares:
Image analysis: ImageJ-Fiji (version 1.53, https://imagej.nih.gov/), LAS X (version 3.7.4.23463 LeicaMicrosystems).
scRNA seq data: Seurat 4 in R 4.0.5, GSEA (http://software.broadinstitute.org/gsea/index.jsp)
Flow Cytometry: BD FACSDiva 8.0.1, FlowingSoftware 2.5.1.
Behaviour: Ethovision XT 11
Electrophysiology: in vivo , Sirenia seizure (v1.7, Pinnacle), ex vitro, Clampex and Clampfit (v10.3)
statistic, analysis and graphs: Origin Pro (2022, Origin Lab), Imaris v9.1.1 (Bitplane), Python (Python.org), GraphPad Prism 9 (GraphPad), Adobe Illustrator and Adobe photoshop (Adobe 2023), Microsoft excel, Matlab 2019b, Spike2 version 8
Resources used for custom Python v3.7.6 virtual environment code:
Jupyter Notebook http://jupyter.org (6.4.12)
NumPy http://www.numpy.org (NumPy v1.19.5)
Scikit https://scikit-image.org (v1.2.0)
Holoviews https://holoviews.org/ (1.15.4)
Neurokit2 https://neurokit2.readthedocs.io/en/latest (0.1.6)
Scipy.Signal (SciPy v1.10.0) Virtanen et al., 2020 Nature Methods; https://docs.scipy.org/doc/scipy/index.html

Details of the software codes used are described in the method section and their availability is as stated in the "Code availability" section in the manuscript
HDF5Array_1.28.1 ; rhdf5_2.44.0 ; DelayedArray_0.26.3 ; S4Arrays_1.0.4; patchwork_1.1.2; reticulate_1.28 ; Matrix_1.5-4.1; cowplot_1.1.1; ggExtra_0.10.0; ggplot2_3.4.2; dplyr_1.1.2; wesanderson_0.3.6 ; RColorBrewer_1.1-3; Seurat_4.9.9.9042; SeuratObject_4.9.9.9084; bmrm_4.4; SummarizedExperiment_1.30.1; Biobase_2.60.0; GenomicRanges_1.52.0; GenomeInfoDb_1.36.0; IRanges_2.34.0 ;

S4Vectors_0.38.1 ; BiocGenerics_0.46.0 ; MatrixGenerics_1.12.0 ; matrixStats_0.63.0 ; torch_0.10.0
Details of the software codes used are described in the method section and their availability is as stated in the "Code availability" section in the manuscript

For manuscripts utilizing custom algorithms or software that are central to the research but not yet described in published literature, software must be made available to editors and reviewers. We strongly encourage code deposition in a community repository (e.g. GitHub). See the Nature Portfolio guidelines for submitting code & software for further information.

Data

Policy information about availability of data

All manuscripts must include a data availability statement. This statement should provide the following information, where applicable:

- Accession codes, unique identifiers, or web links for publicly available datasets
- A description of any restrictions on data availability
- For clinical datasets or third party data, please ensure that the statement adheres to our policy

We have included a data availability statement in the manuscript. Notably, the new single-cell RNAseq datasets generated during and/or analyzed during the current study are made available for download on Zenodo repository (10.5281/zenodo.7704838). The already published and available datasets analyzed during the current study and their public domain resources are indicated in Extended Figure 1a and in Methods:
GSE106447 “Artegiani” – https://doi.org:10.1016/j.celrep.2017.11.050;
GSE114000 “Batiuk” - https://doi.org:10.1038/s41467-019-14198-8;
GSE143758 "Habib" - https://doi.org:10.1038/s41593-020-0624-8;
GSE95753 “Hochgerner” - https://doi.org:10.1038/s41593-017-0056-2;
SRP135960 “Zeisel-1” https://doi.org:https://doi.org/10.1016/j.cell.2018.06.021;
“Saunders” - http://dropviz.org;
GSE60361 “Zeisel-2” - https://doi.org:doi:10.1126/science.aaa1934;
“Yao” - https://portal.brain-map.org/atlases-and-data/rnaseq/mouse-whole-cortex-and-hippocampus-10x
“Habib Human” - https://www.gtexportal.org/home/datasets
GSE160189 “Ayhan” - https://doi.org:10.1016/j.neuron.2021.05.003;
“Tran” - https://github.com/LieberInstitute/10xPilot_snRNAseq-human
GSE190940 “Zipursky” - https://doi.org/10.1016/j.cell.2021.12.022;
EMBL-EBI repository E-MTAB-10459 “Liu” - https://www.ebi.ac.uk/biostudies/arrayexpress/studies/E-MTAB-10459;
GSE97930 “Zhang” - https://doi.org:10.1038/nbt.4038;
GSM4157078 “Agarwal” - https://doi.org:10.1038/s41467-020-17876-0;
GSE126836 “Welch” - https://doi.org:https://doi.org/10.1016/j.cell.2019.05.006.

Field-specific reporting

Please select the one below that is the best fit for your research. If you are not sure, read the appropriate sections before making your selection.

☒ Life sciences ☐ Behavioural & social sciences ☐ Ecological, evolutionary & environmental sciences

For a reference copy of the document with all sections, see nature.com/documents/nr-reporting-summary-flat.pdf

Life sciences study design

All studies must disclose on these points even when the disclosure is negative.

Sample size	The nature of the n is described for each experiment in the corresponding figure legends. Sample size determinations are based on previous experience (Habbas et al., Cell, 2015, XDi Castro et al., Nat. Neurosic., 2011) and standards in the field (Rusina et al.,eNeuro,2021; D'Amour etal. Exp. Neurol.,2015). The low variability between the same type of samples, as indicated by the SEM, confirms that the sampling was sufficient to observe statistically significant differences between groups. Where variability was expected to be higher (such as for instance in in vivo experiments), up to 13 biological replicates were included.
Data exclusions	Patchseq: low quality RNAseq cells were eliminated as described in the method section.<br>EEG recording: mice that detached from the recording system during the experiments or died after KA administration were excluded.<br>Behavior: mice that in the Inter E-shock Interval 5-6 did not reach 40% of freezing or went over 70% were excluded as used in Contextual fear conditioning experiments. This range represents the optimal window for being in the position to observe either increments or decrements in performance potentially induced by genetic or pharmacological interference.<br>Glutamate imaging analysis: FOV that did not respond to glutamate as positive control were excluded.<br>in vivo fiber photometry fluorescence measurements: excluded mice that have no stable signal during the baseline experiment<br>in vivo two-photon experiments: FOV with artifacts due to mouse movements were excluded. for details see in vivo imaging "Methods" section
Replication	Experiments were repeated as indicated in detail for each figure panel and as described in the methods.<br>Reported findings were reproduced across animals in EEG recordings, behavioural and microdialysis experiments as well as across cells/ animals in patchseq, imaging and electrophysiology experiments.<br>In patchseq experiments, each single-cell RNA sequencing pool contained cells from different collection days and conditions to minimize batch effect.<br>The total number of animals and cells/FOV is reported for all experiments. The replications are shown as individual dots together with the

calculated means and variability (mean +/- sem).

Randomization	In all experiments using littermate mice with different pharmacological treatments, animals were randomized in the various groups to avoid cage, litter and batch effects.
Blinding	Data acquisition and analysis were done blind when possible. Cell clusters, based on gene expression patterns, were identified computationally without input from a trained neuroscientist. Independent researchers were 'blinded' to each others work.

Reporting for specific materials, systems and methods

We require information from authors about some types of materials, experimental systems and methods used in many studies. Here, indicate whether each material, system or method listed is relevant to your study. If you are not sure if a list item applies to your research, read the appropriate section before selecting a response.

Materials & experimental systems

n/a	Involved in the study
☐	☒ Antibodies
☒	☐ Eukaryotic cell lines
☒	☐ Palaeontology and archaeology
☐	☒ Animals and other organisms
☒	☐ Human research participants
☒	☐ Clinical data
☒	☐ Dual use research of concern

Methods

n/a	Involved in the study
☒	☐ ChIP-seq
☐	☒ Flow cytometry
☒	☐ MRI-based neuroimaging

Antibodies

Antibodies used

All antibodies (provider, ordering numbers and dilution) used in this study are listed in supplementary Table 3.

guine pig anti-Iba1 Synaptic System 234004 2-28 1:500
mouse anti-Cre Recombinase Merk millipore MAB3120 JC1631396 1:500
mouse anti-S-100b SIGMA S2532 048m4858v 1:500
mouse anti-NeuN Merk millipore MAB377 1991263 1:500
mouse anti-glutamine synthetase Merk millipore MAB302 2676275 1:500
mouse anti-Olig2 Merk millipore MABN50 3421971 1:500
rabbit anti-glutamine synthetase Abcam ab 73593 gr3200078-1 1:500
rabbit anti-S100b Synaptic system S287003 1-5 1:500
rabbit anti-Olig2 NovusBio NBP1-28667 DF1210 1:100
rabbit Anti-Tyrosine Hydroxylase Merk millipore AB152 3114503 1:200
chicken anti-S100b Synaptic system 287006 287006/1-4 1:500
goat anti-TdTomato BioSource cat. n. MBS448092 lot: 0081191218 1:500

Goat anti-Mouse IgG (H+L) Highly Cross-Adsorbed Secondary Antibody, Alexa Fluor Plus 405 Thermofisher Cat # A48255 51912A 1:500
Goat anti-Mouse IgG (H+L) Highly Cross-Adsorbed Secondary Antibody, Alexa Fluor Plus 488 Thermofisher Cat # A-32723 TC252656 1:500
Goat anti-Mouse IgG (H+L) Highly Cross-Adsorbed Secondary Antibody, Alexa Fluor 633 Thermofisher Cat # A-21052 1906490 1:500
Goat anti-Rabbit IgG (H+L) Highly Cross-Adsorbed Secondary Antibody, Alexa Fluor Plus 488 Thermofisher Cat # A-11034 SH251139 1:500
Goat anti-Rabbit IgG (H+L) Highly Cross-Adsorbed Secondary Antibody, Alexa Fluor 633 Thermofisher Cat # A-21071 1932492 1:500
Goat anti-Guinea Pig IgG (H+L) Highly Cross-Adsorbed Secondary Antibody, Alexa Fluor 488 Thermofisher Cat # A-11073 1458631 1:500
Goat anti-Guinea Pig IgG (H+L) Highly Cross-Adsorbed Secondary Antibody, Alexa Fluor 633 Thermofisher Cat # A-21105 514962 1:500

Validation

All antibodies used in this study are from commercial suppliers (see notes above for each antibody) that have verified the specificity of the antibodies. All the antibodies have been previously used by various laboratories. All secondary antibodies are verified to not give a specific staining without the primary antibody.

Primary antibodies:
mouse anti-Cre Recombinase Merck millipore MAB3120 JC1631396 1:500 - Manufacturer:Proven to reliably detect Cre Recombinase, this mAb is validated for use in ELISA, IC, IF, IH & WB and is backed by multiple publications.
mouse anti-S-100b SIGMA S2532 048m4858v 1:500 -Manufacturer:These antibodies have been verified by Relative Expression to confirm specificity to S100B.
mouse anti-NeuN Merck millipore MAB377 1991263 1:500 - Manufacturer: it detects level of NeuN and has been published and validated for use in FC, IC, IF, IH, IH(P), IP and WB. Positive control -Brain Tissue. Negative control - Any non neuronal tissue eg Fibroblasts
mouse anti-glutamine synthetase Merck millipore MAB302 2676275 1:500 -Manufacturer: Detect Glutamine Synthetase using this Anti-Glutamine Synthetase Antibody, clone GS-6 validated for use in ELISA, IH, IH(P) & WB with more than 45 product citations.Controlled in Rat brain tissue, rat brain cytosolic fraction extract
mouse anti-Olig2 Merk millipore MABN50 3421971 1:500 - Manufacturer:clone 211F1.1, from mouse.controlled on mouse brain

samples.
rabbit anti-glutamine synthetase Abcam ab 73593 gr3200078-1 1:500 -Manufacturer:Suitable for: IHC-P, ICC/IF, WB. Species reactivity: Mouse, Rat, Human, Common marmoset.
rabbit anti-S100b Synaptic system S287003 1-5 1:500 -Manufacturer: Reacts with: rat (P04631), mouse (P50114).
rabbit anti-Olig2 NovusBio NBP1-28667 DF1210 1:100 -Manufacturer: Reacts with: human, rat, mouse. Validated for use in IH and IH(P) with more than 25 product citations.
rabbit Anti-Tyrosine Hydroxylase Merk millipore AB152 3114503 1:200 -Manufacturer:validated for use in ELISA, IH, IH(P) & WB with more than 35 product citations.Controlled in Human, Rat, Mouse, Drosophila,Cat, Ferret, Squid, Mollusc.
chicken anti-S100b Synaptic system 287006/1-4 1:500 -Manufacturer: Reacts with: rat (P04631), mouse (P50114). Validated for use in IHP, ICC,IHC.
goat anti-TdTomato BioSource cat. n. MBS448092 lot: 0081191218 1:500 -Manufacturer:validated for use in IF, IHC-P, IHC-F & WB

Secondary antibodies:
Goat anti-Mouse IgG (H+L) Highly Cross-Adsorbed Secondary Antibody, Alexa Fluor Plus 405 Thermofisher Cat # A48255 51912A 1:500
Goat anti-Mouse IgG (H+L) Highly Cross-Adsorbed Secondary Antibody, Alexa Fluor Plus 488 Thermofisher Cat # A-32723 TC252656 1:500
Goat anti-Mouse IgG (H+L) Highly Cross-Adsorbed Secondary Antibody, Alexa Fluor 633 Thermofisher Cat # A-21052 1906490 1:500
Goat anti-Rabbit IgG (H+L) Highly Cross-Adsorbed Secondary Antibody, Alexa Fluor Plus 488 Thermofisher Cat # A-11034 SH251139 1:500
Goat anti-Rabbit IgG (H+L) Highly Cross-Adsorbed Secondary Antibody, Alexa Fluor 633 Thermofisher Cat # A-21071 1932492 1:500
Goat anti-Guinea Pig IgG (H+L) Highly Cross-Adsorbed Secondary Antibody, Alexa Fluor 488 Thermofisher Cat # A-11073 1458631 1:500
Goat anti-Guinea Pig IgG (H+L) Highly Cross-Adsorbed Secondary Antibody, Alexa Fluor 633 Thermofisher Cat # A-21105 514962 1:500
Alexa Fluor-568 dk anti-goat IgG H+L Invitrogen cat. n. A11057 2421197 1:500
Alexa Fluor Plus-647 dk anti-mouse IgG H+L Invitrogen cat. n. A32787 TJ271040 1:500
guine pig anti-Iba1 Synaptic System 234004 2-28 1:500 - Manufacturer: Reacts with: mouse (Q9EQW9), rat (P55009), human (P55008).
To minimize cross-reactivity, Highly Cross-adsorbed secondary antibodies where preferred. These antibodies have been highly cross-adsorbed against bovine IgG, goat IgG, mouse IgG, rat IgG, and human IgG. Cross-adsorption or pre-adsorption is a purification step to increase specificity of the antibody resulting in higher sensitivity and less background staining. The secondary antibody solution is passed through a column matrix containing immobilized serum proteins from potentially cross-reactive species. Only the nonspecific-binding secondary antibodies are captured in the column, and the highly specific secondaries flow through. Further passages through additional columns result in 'highly cross-adsorbed' preparations of secondary antibody. The benefits of these extra steps are apparent in multiplexing/multicolor-staining experiments where there is potential cross-reactivity with other primary antibodies or in tissue/cell fluorescent staining experiments where there may be the presence of endogenous immunoglobulins.
Alexa Fluor dyes are among the most trusted fluorescent dyes available today.

Simultaneous staining for the following sets of antibodies were performed to validate cross-identification of the same protein:
-mouse, rabbit and chicken anti-S100b
-mouse and rabbit Glutamine Synthetase
-mouse and rabbit anti Olig2

Animals and other organisms

Policy information about studies involving animals; ARRIVE guidelines recommended for reporting animal research

Laboratory animals

original mouse lines used to generate the mouse lines used in this study:
hGFAP-CreERT2, Tg(GFAP-cre/ERT2)1Fki, ref. https://onlinelibrary.wiley.com/doi/10.1002/glia.20342
Slc17a6fl/fl, ref. https://www.ncbi.nlm.nih.gov/pmc/articles/PMC2846457/
Slc17a7fl/fl, https://doi.org:10.1101/2021.06.19.449108 ref.
GLASTcreERT2P2ry1fl/fl https://doi.org:https://doi.org/10.1002/glia.22999
Rosa26-loxP-stop-loxP-tdTomato reporter mice (Ai14), Jackson Stock No: 007908 - B6;129S6-Gt(ROSA)26Sortm14(CAG-tdTomato)Hze/J
B6;-Gt(ROSA)26Sortm95.1(CAG-GCaMP6f)Hze/J, JAX 024105
C57/Bl6 mice, Janvier, France

derived mouse lines were used in this study:
33 male 2-4 months C57/Bl6 mice, Janvier, France
27 male 1-3 months hGFAP-CreERT2, Rosa26-loxP-stop-loxP-tdTomato
105 male 1-5 months hGFAP-CreERT2, Slc17a7fl/fl,Rosa26-loxP-stop-loxP-tdTomato
114 male 1-3 months hGFAP-CreERT2, Slc17a6fl/fl,Rosa26-loxP-stop-loxP-tdTomato
2 male 4-5 months GLASTcreERT2P2ry1fl/fl
2 male 4-5 months hGFAP-CreERT2, Rosa26-loxP-stop-loxP-GCaMP6f

Housing conditions were as following: dark/light cycle 12/12, ambient temperature around 21-22°C and humidity between 40 and 70% (55% in average).

Wild animals

The study did not involve wild animals.

Field-collected samples

The study did not involve field-collected samples

Ethics oversight

All experiments including animals were carried out in compliance with the Swiss Federal and Cantonal authorities (authorizations:

Ethics oversight	VD1873.1, VD2982, VD3053.1, VD3115.1) or by the Council Directive of the European Communities (2010/63/EU), and the Animal Care Committee of Italian Ministry of Health (authorization: 375/2018-PR).

Note that full information on the approval of the study protocol must also be provided in the manuscript.

Flow Cytometry

Plots

Confirm that:

☒ The axis labels state the marker and fluorochrome used (e.g. CD4-FITC).

☒ The axis scales are clearly visible. Include numbers along axes only for bottom left plot of group (a 'group' is an analysis of identical markers).

☒ All plots are contour plots with outliers or pseudocolor plots.

☒ A numerical value for number of cells or percentage (with statistics) is provided.

Methodology

Sample preparation	the detailed step-by-step procedure for preparation of a single-cell suspension from mouse brain regions is described in the method section.
Instrument	BD FACS Aria III
Software	BD FACSDiva software (BD Biosciences) and FlowingSoftware (TreeStar)
Cell population abundance	Purity checked of tdtomato sorted cells fraction was 88-98%.
Gating strategy	Cells were gated on forward/side scatter, live/dead by DAPI exclusion, and tdTomato (BP 585/42), using tdTomato and DAPI controls to set gates for each experiment. See Extended Figure 8 and 9.

☒ Tick this box to confirm that a figure exemplifying the gating strategy is provided in the Supplementary Information.
